# Functional near-infrared spectroscopy short-channel regression improves cortical activation estimates of working memory load

**DOI:** 10.1117/1.NPh.12.3.035009

**Published:** 2025-08-21

**Authors:** Jiahui An, Pulkit Goyal, Andreas R. Luft, Josef G. Schönhammer

**Affiliations:** a University of Zürich, ETH Zürich, Institute of Neuroinformatics, Zürich, Switzerland; b University of Tübingen, Graduate Training Center of Neuroscience, Tübingen, Germany; c University Hospital Zürich, University of Zürich, Division of Vascular Neurology and Neurorehabilitation, Department of Neurology and Clinical Neuroscience Center, Zürich, Switzerland; d Lake Lucerne Institute, NeuroCoRe Lab, Vitznau, Switzerland

**Keywords:** working memory load, short separation channels, functional near-infrared spectroscopy, brain–computer interface, *N*-Back, mental workload

## Abstract

**Significance:**

Functional near-infrared spectroscopy (fNIRS) is a noninvasive technique commonly used to examine cognitive functions such as working memory (WM). However, fNIRS signals are often interfered with by extracerebral activity, such as scalp hemodynamics. Short separation channels (SSCs) allow direct measurement of these signals. Short-channel regression (SCR) is widely used to reduce scalp interference, but its added value in WM paradigms remains underexplored.

**Aim:**

We aimed to examine the effect of SCR on improving the validity of fNIRS measurements for WM load (WML).

**Approach:**

We used the N-Back task to induce WML-dependent brain activation by varying the “n” level. Data from 20 participants were collected using fNIRS with SSC. Hemodynamic responses were analyzed with generalized linear models and linear mixed models to assess SCR’s effect on the sensitivity of cortical activation measures.

**Results:**

SCR enhanced the statistical effects of N-Back levels on measured hemodynamic responses at both group and subject levels, improving the validity and sensitivity of fNIRS.

**Conclusions:**

SCR improves fNIRS measurement sensitivity and validity, even in tasks with minimal motor requirements.

## 1 Introduction

Functional near-infrared spectroscopy (fNIRS) is becoming more prominent as a neuroimaging technique. It is noninvasive, uses low-intensity near-infrared (NIR) light to measure brain activity, and is more tolerant to motion and electrical interference compared with electroencephalography (EEG), functional magnetic resonance imaging (fMRI), and positron emission tomography (PET). These properties make fNIRS well suited for studying populations who cannot stay still, individuals with ferromagnetic implants, or conducting research in applied settings.^1^^–^^4^

The versatility of fNIRS is based on the relative transparency of human tissue to near-infrared (NIR) light. NIR light penetrates several centimeters into tissue, where it is absorbed and scattered. Oxygenated hemoglobin (HbO) and deoxygenated hemoglobin (HbR) absorb NIR light differently, enabling the detection of concentration changes linked to neuronal and nonneuronal activity.^5^^,^^6^ Specifically, brain activation typically induces localized increases in cerebral blood flow, resulting in increased HbO and decreased HbR concentrations—a process known as neurovascular coupling, although its underlying mechanisms are still a topic of ongoing research and debate.^7^

Despite its advantages, fNIRS measurements can still be influenced by superficial physiological activity such as influences from the scalp and systemic physiological changes.^5^^,^^8^^–^^14^ Investigation of the activity in short separation channels (SSCs, typically 8 mm apart) that mainly capture scalp signals showed that incidental body movements can exaggerate hemoglobin concentration changes during motor tasks^9^^,^^10^^,^^15^^–^^17^ and that varying levels of vocalization can confound the results of language tasks.^11^ Critically, these influences could lead to false-positive or false-negative findings in the observed cerebral hemodynamic responses.^5^^,^^8^^,^^18^

To mitigate scalp-related noise, short-channel regression (SCR) has been proposed.^5^^,^^8^^,^^19^^–^^22^ SCR uses SSCs to measure superficial hemodynamics and regress these out from long-separation channels that capture both cortical and scalp signals.^5^^,^^8^^,^^15^^,^^23^^–^^25^ Each SSC acts as a local regressor for nearby long channels, accounting for regional variability in physiological noise.^26^ Numerous studies have shown that SCR effectively reduces extracerebral interference,^10^^,^^11^ improving key statistical metrics such as t-values,^25^ contrast-to-noise ratio,^27^ and the detection rate of active channels.^28^

fNIRS has been widely used to investigate working memory (WM) processes.^29^^–^^40^ WM is a core cognitive process that enables the temporary storage and manipulation of information,^41^ thus forming the basis for many higher order cognitive processes.^42^ To investigate WM, fNIRS studies have frequently employed variants of the N-Back task,^29^^–^^35^^,^^40^ in which participants identify whether the current stimulus matches one presented n steps earlier. The N-Back task is designed under the assumption that higher N-Back levels require more storage and manipulation, leading to increased working memory load (WML).^43^^–^^45^ Consistent with this expectation, the mentioned fNIRS studies observed that the increasing N-Back levels were associated with increased HbO and decreased HbR concentration changes in prefrontal regions. These findings align with previous fMRI and PET studies, which demonstrated that increasing N-Back levels led to greater activation in the dorsolateral prefrontal cortex (DLPFC) and the posterior parietal cortex (PPC).^40^^,^^46^^–^^50^

However, the aforementioned fNIRS studies using the N-Back task did not apply SCR to mitigate superficial physiological noise.^29^^–^^35^^,^^40^ One study measured extracerebral signals peripherally and found it effective in modeling and removing nonneuronal influences from fNIRS signals, but it did not use SCR from SSCs.^40^ Although SCR has been extensively validated in motor, pain, and semantic memory paradigms,^51^^–^^53^ its application in cognitive tasks with minimal motion, such as the N-Back task, remains relatively underexplored. This gap is important to address as evidence suggests that even in low-motion tasks, superficial physiological signals can confound fNIRS results.^11^^,^^54^^,^^55^

Therefore, our study aimed to evaluate whether scalp-level confounds influence fNIRS measurements during a standard cognitive task with minimal motor requirements, such as the N-Back, and whether SCR can still enhance the sensitivity and validity of the resulting signals. Specifically, we used the N-Back task to manipulate WML and tested three hypotheses. First, we hypothesized that increasing N-Back levels would result in greater activation in the PFC and PPC, reflected by increased HbO and decreased HbR concentrations. Second, we hypothesized that applying SCR would enhance the ability to distinguish between load levels by improving the statistical robustness of fNIRS results, measured by higher t-values and a greater number of significant channels. Third, we hypothesized that the effects of WML observed at the group level would be reflected consistently at the individual subject level when using SCR. In summary, we aimed to demonstrate that SCR improves fNIRS sensitivity and statistical robustness in well-controlled cognitive paradigms where motion-related confounds are minimal.

## 2 Method

### 2.1 Participants

A total of 22 healthy young individuals participated in this study. All had normal or corrected-to-normal vision and no cognitive or orthopedic disabilities. Two participants were excluded due to malfunction of the acquisition system, which resulted in abnormal signal patterns (i.e., HbO was consistently negative and HbR positive). This left a final group of 20 participants (10 males and 10 females). The average age was 25.4 years with a range of 21 to 28 years. With respect to ethnicity, one participant was of African descent, one of Middle Eastern descent, one of Asian descent, and the remaining participants were of European descent.

This study was conducted in accordance with the principles of the Declaration of Helsinki. The study was approved by the ETH Ethics Committee (2018-N-22) and the Cantonal Ethics Commission of the Canton of Zurich (2018-01078). All participants gave written informed consent before the study and participated voluntarily.

### 2.2 Experimental Paradigm and Procedure

The experimental paradigm consisted of a N-Back task with four conditions, as described by Braver et al.^47^ and Veltman et al.^48^ We closely followed the experimental design of Braver et al., whose study we used to guide the optode placement in our fNIRS setup. This approach was chosen to maximize the likelihood of replicating their reported WML effects in similar brain areas. The task was presented on a Dell laptop with a 14-in. screen. Stimulus presentation and behavioral data collection were conducted using MATLAB 2022b and Psychtoolbox-3.^56^ For synchronization, trigger messages were sent from the stimulus presentation to an Arduino Nano, which sent impulses to the fNIRS system.

**Fig. 1:**
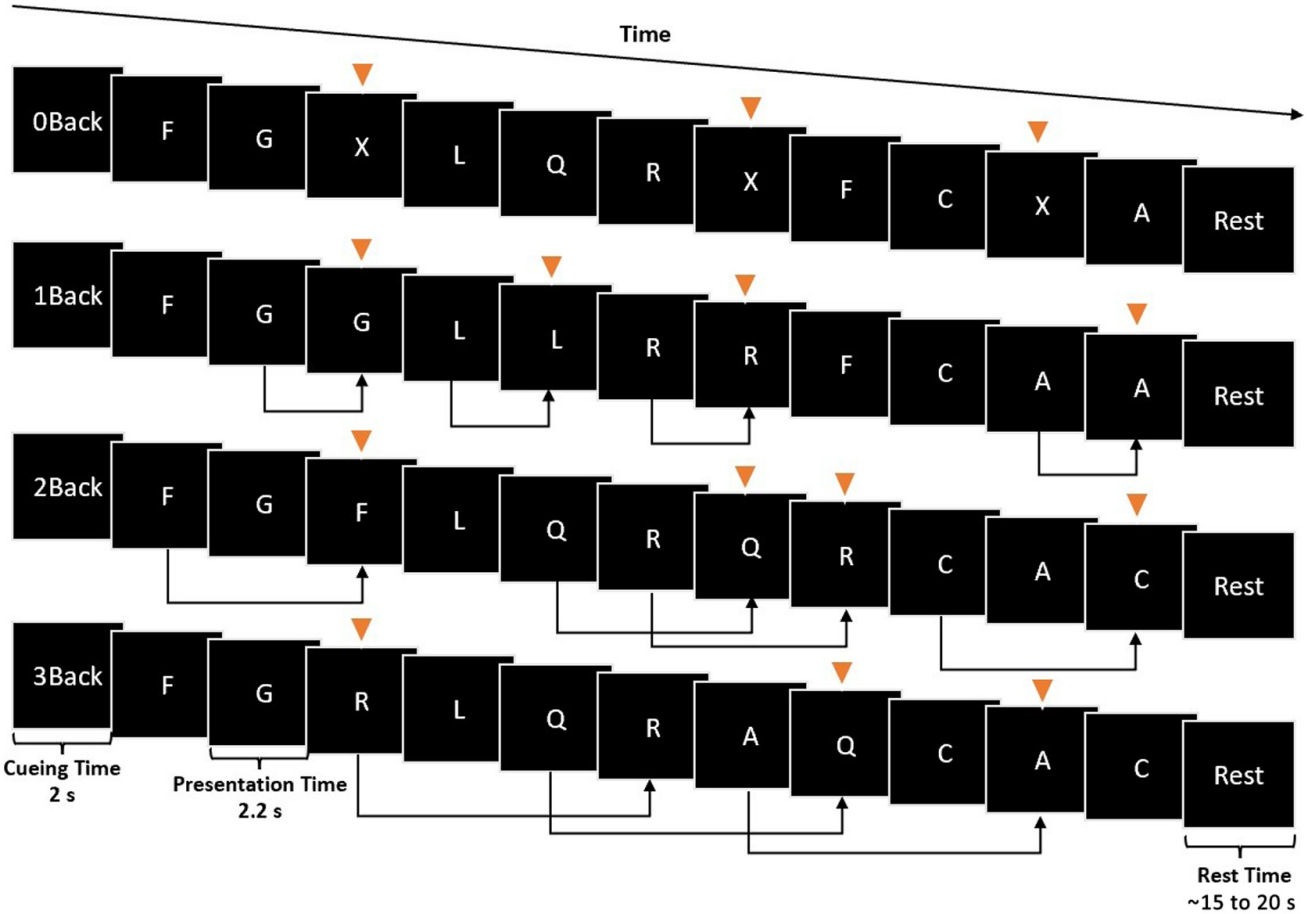
Illustration of the displays shown in an individual block of the different N-Back levels of the experimental task. The block start, the N-Back level of the block, was presented. Each letter stimulus was presented for 2.2 s. For illustrative purposes, the figure includes orange triangles to indicate the targets. In the 0-Back condition, targets were instances of the letter “X”. For the 1-Back to 3-Back conditions, targets were letters that matched the letter presented N-Back steps before the current letter, as illustrated by black arrows. Participants were instructed to press one key when they identified a target letter and a different key for nontarget letters. Thus, correct responses required participants to maintain different lengths of letter sequences in WM, varying the level of WML. After each block, a rest interval of 15 to 20 s occurred.

The experimental task was divided into blocks, with each block presenting one of the four N-Back conditions, as illustrated in Fig. 1. Each block began with a 0.2-s sound signaling its start, followed by a 2-s interval indicating the N-Back level of the upcoming block. Then, 10 capitalized letters, randomly drawn from the English consonants with replacement, were presented.

For the 0-Back condition, targets were instances of the letter “X.” For the 1-Back to 3-Back conditions, targets were letters that matched the letter presented N-Back steps before the current letter. Within each block of 10 letters, three to four were targets. Each stimulus was displayed for 2.2 s, followed by a 0.7-s blank interval during which participants responded. Participants pressed the “F” key with their left index finger to indicate the targets and the “J” key with their right index finger to indicate nontargets. Then, the 0.3-s feedback was provided to indicate whether the response was correct or incorrect. We provided immediate feedback after each response to support participants in maintaining high accuracy and to minimize speed-accuracy trade-offs.

The experimental procedure, conducted individually for each participant, involved the following steps. At the start of a session, participants were seated at a desk in front of a laptop in a normally lit room. After providing informed consent, participants received both written and oral explanations of the task and then practiced each N-Back level. During these practice trials, real-time feedback was provided after each response, indicating whether the response was correct, incorrect, early (response time <0.1  s), or missing, to familiarize participants with the task.

Next, the fNIRS cap was positioned on participants’ heads, and target ROIs were localized. Sensors were mounted in the holders of the cap, and each source’s and detector’s location was registered. The fNIRS equipment was calibrated before data recording. Suboptimal channels were adjusted by repositioning optodes and/or removing hair from the sensor sites, followed by recalibration.

The experiment began with a 1-min resting period during which participants fixated on a central cross. Following this, participants completed 40 blocks of the N-Back tasks. To control for order effects, we employed a 4×4 Latin square to counterbalance the sequence of four N-back levels across participants. Each participant was randomly assigned to one of the four rows of the Latin square, ensuring that each condition appeared exactly once in each serial position and was equally likely to follow any other condition across the experiment. After each block, a variable 15- to 20-s resting period with a central fixation cross was included to allow the hemodynamic response to return to baseline. The task procedure lasted between 36 and 38 min, with the overall study duration up to 70 min per participant.

### 2.3 fNIRS System

fNIRS data were obtained using a continuous-wave fNIRS system prototype (optohive^57^) with LED sources emitting light at 735 and 850 nm. Each module contained one light source and one detector positioned 7.5 mm apart, forming an embedded SSC for measuring superficial hemodynamics. In addition, long-separation channels were created by pairing sources and detectors across different modules spaced ∼30  mm apart.

As several optode batteries became nonfunctional over time, both the sampling frequency and the number of available optodes varied across participants. The sampling frequency ranged from 9.85 to 14.08 Hz. The number of optodes used per participant was as follows: 6 optodes for 1 participant, 7 for 2 participants, 8 for 4 participants, 9 for 8 participants, 10 for 1 participant, and 11 for 4 participants. Depending on the participant’s montage, this configuration yielded between 9 and 15 long-separation channels. These channels were arranged to target working memory-related regions in the PFC and parietal cortices, as detailed in Sec. 2.4 and Table 1. The overall optode layout and corresponding long-channel layout are shown in Figs. 3(a) and 3(b).

**Table 1:** fNIRS channels, their MNI coordinates, related brain parcellation areas, and specificity of targeting these brain areas. The brain areas contained those reported in previous fMRI to be associated with WML in the N-Back task.^47^^,^^48^

Ch#	X	Y	Z	Anatomical region	Specificity
1	−23	26	56	L middle frontal gyrus	46.014
	−23	26	56	L superior frontal gyrus	45.930
2	−38	12	55	L middle frontal gyrus	66.609
	−38	12	55	L precentral gyrus	25.716
3	−38	12	55	L middle frontal gyrus	66.609
	−38	12	55	L precentral gyrus	25.716
	−38	12	55	L superior frontal gyrus	6.504
4	−31	39	41	L middle frontal gyrus	68.057
	−31	39	41	L superior frontal gyrus	30.841
5	−45	25	41	L middle frontal gyrus	81.084
	−45	25	41	L precentral gyrus	9.360
6	−51	−3	48	L precentral gyrus	54.847
	−51	−3	48	L postcentral gyrus	36.779
7	−55	12	34	L precentral gyrus	47.781
	−55	12	34	L IFG (p. Triangularis)	18.150
	−55	12	34	L IFG (p. Opercularis)	12.640
	−55	12	34	L middle frontal gyrus	12.112
8	−51	−3	48	L precentral gyrus	54.847
	−51	−3	48	L postcentral gyrus	36.779
9	−45	26	41	L middle frontal gyrus	81.900
	−45	26	41	L IFG (p. Triangularis)	9.104
10	−62	−3	22	L postcentral gyrus	55.769
	−62	−3	22	L precentral gyrus	14.854
	−62	−3	22	L rolandic operculum	10.476
11	−56	24	20	L IFG (p. Triangularis)	71.116
	−56	24	20	L IFG (p. Opercularis)	16.467
12	−59	9	7	L IFG (p. Opercularis)	25.259
	−59	9	7	L rolandic operculum	21.029
	−59	9	7	L superior temporal gyrus	17.256
	−59	9	7	L IFG (p. Triangularis)	16.134
13	−39	−49	60	L inferior parietal lobule	38.116
	−39	−49	60	L superior parietal lobule	31.683
	−39	−49	60	L postcentral gyrus	26.065
14	−53	−34	51	L inferior parietal lobule	57.998
	−53	−34	51	L postcentral gyrus	29.701
	−53	−34	51	L supra marginal gyrus	10.628
15	−57	−47	37	L inferior parietal lobule	40.670
	−57	−47	37	L supra marginal gyrus	36.749
	−57	−47	37	L angular gyrus	16.992

### 2.4 fNIRS Optode Locations

The locations of the optodes were aligned with brain regions reported to be load-sensitive during N-Back tasks, as identified in previous fMRI studies.^47^^,^^48^ To enable use with the TMS-based Brainsight neuronavigation system—which accepts only MNI coordinates—we converted the Talairach coordinates into the Montreal Neurological Institute (MNI) space using the tal2mni.m function.^58^ Specifically, we utilized the Talairach coordinates reported for the DLPFC (−36 48 21 and −45 36 27), the Ventrolateral Prefrontal Cortex (VLPFC) (−51 12 9), and the Posterior Parietal Cortex (PPC) (−45 −39 51).^48^ The transformed MNI coordinates were (−36.4 48.3 25.4 and −45.5 35.7 31.3) for DLPFC, (−51.5 11.9 10.4) for VLPFC, and (−45.5 −42.8 53.2) for PPC.

For each participant, the MNI coordinates were localized and projected to the scalp using a Brainsight TMS Neuronavigation system and Brainsight TMS neuronavigation software, v2.3 (Rogue Research, Canada). Optodes were then placed to cover the scalp locations above these coordinates, and source and detector locations were registered with the Brainsight system, yielding coordinates in the MNI coordinate system. Given the limited number of optodes, we positioned them exclusively in the left hemisphere, as shown in Fig. 3(a), based on the left-lateralized brain activation observed in the aforementioned studies. Three optodes were designated for the parietal cortex, whereas the remainder were allocated to the prefrontal cortex. To identify the underlying brain area of each channel, each channel’s coordinators were mapped to a brain parcellation atlas (Colin2) using (fold_channel_specificity(), MNE-NIRS).^13^

The corresponding brain landmarks for each channel based on the fOLD toolbox were listed in Table 1 together with mean MNI coordinates across all participants, the corresponding brain regions, and the specificity for each channel. Specificity refers to the percentage of each channel’s overlap with the mentioned brain region.^13^

### 2.5 Analysis

#### 2.5.1 Behavioral data

Behavioral data were analyzed using MATLAB 2022b. For each participant, response accuracy in a block was calculated by the proportion of correct responses in the block. Then, for each N-Back level, accuracy was averaged across the 10 blocks of the level. For each participant, response times (RTs) were averaged across the correct and incorrect trials of each N-Back level to inspect the speed-accuracy trade-off.^59^ One-way repeated-measures ANOVAs with the factor N-Back level were used to analyze mean accuracy and RTs, with eta squared ($\eta^{2}$) quantifying the effect size. Significant ANOVA results were followed by pairwise comparisons using Benjamini–Hochberg false-discovery rate (FDR) corrected paired t-tests. Cohen’s d was calculated for each pairwise comparison to determine the effect size.

#### 2.5.2 fNIRS data: preprocessing, generalized linear model (GLM), and linear mixed models (LMMs)

MNE toolbox,^60^^,^^61^ MNE-NIRS Toolbox,^28^ and fnirsPy^62^ were used for the analysis of fNIRS data. The MNE and MNE-NIRS toolbox are Python toolboxes for analyzing electrophysiological and fNIRS data. fnirsPy is a wrapper that integrates the MNE and MNE-NIRS toolboxes and facilitates preprocessing of fNIRS data.

The fnirsPy toolbox provided a preprocessing pipeline including several steps. Ambient light adjustment (ambient light removal) was performed using polynomial regression, as implemented by (remove_backlight(), fnirsPy). We chose polynomial regression because the ambient light in our recordings exhibited slow, nonlinear drifts over time, which this method effectively captures and corrects. Then, raw intensity was converted to optical density using (optical_density(), MNE-NIRS). Motion artifact correction was based on the (temporal_derivative_distribution_repair(), MNE-NIRS), which uses the temporal derivative distribution repair (TDDR) method. TDDR identifies abrupt signal changes by analyzing the temporal derivative distribution of each fNIRS channel. It iteratively down-weights outlier derivatives using robust regression to reconstruct a cleaned signal, effectively preserving physiological fluctuations while suppressing motion-induced artifacts.^63^

To evaluate the contact quality of optodes, the scalp coupling index was calculated (scalp_coupling_index(), MNE-NIRS),^64^ and channels with SCI<0.5 were rejected. It was suggested that SCI should be used in combination with a frequency measure of cardiac rhythm.^65^ We employed (autopick_channels(), fnirsPy) in addition to SCI. This function fits a Gaussian curve to the frequency spectrum of optical density between 0.6 and 1.8 Hz and rejects channels with low signal power (0.12 dB), indicating the absence of a cardiac rhythm.

The conversion to HbO and HbR signals was carried out using the modified Beer–Lambert law (beer_lambert_law(), MNE-NIRS). In addition, bandpass filtering (raw.filter(), MNE) was applied in the 0.01 to 0.08 Hz hemodynamic response frequency range using a finite impulse response (FIR) filter with an order of 1000.^66^

The preprocessed HbO and HbR concentration changes were analyzed using GLM analysis. We conducted two separate GLM analyses. Both analyses included regressors for the four N-Back levels (0-Back, 1-Back, 2-Back, and 3-Back), a constant term, and a linear drift model. The linear drift model accounts for low-frequency trends in the data that are unrelated to neural activity (e.g., instrumental drift, baseline shifts, or physiological fluctuations) by fitting a linear function over time and removing its influence on the estimated task-related responses.^67^^,^^68^ In one GLM (GLM-SCR), we included a regressor for SSC activity, which was the mean concentration change of all SSC. In the second GLM (GLM-noSCR), this regressor was omitted. By comparing the results of these two GLMs, we aimed to assess the impact of SCR on the measurement of hemodynamic changes during the N-Back task.

The GLM was implemented using (run_glm(), MNE-NIRS) with the “SPM” canonical hemodynamic response function. Event durations were defined as 35-s intervals, beginning with the cue onset of each block and extending to the block’s end. GLMs’ results were corrected for multiple comparisons using the FDR p-values, using a threshold of p<0.05 across all tests, channels, and N-Back levels. The GLMs’ FDR-adjusted results were sorted by the magnitude of the t-values and annotated with participant IDs. The output of the GLMs included θ-values (as named by the analysis software, these are essentially β-values, the estimated coefficients of the modeled hemodynamic response), t-values, and p-values for both short and long channels in each N-Back level as well as the hemoglobin type. Here, the t-values represent the contrast statistics for each N-Back level condition estimated from the subject-level GLM (e.g., the difference from baseline) and were used as the dependent variables in subsequent LMMs to evaluate group-level effects.

The GLM results were analyzed using linear mixed models (LMMs). The dependent variables were the t-values of the GLM analysis, separately considering those with SCR (GLM-SCR) and without SCR (GLM-noSCR), and those for the HbO and HbR concentration changes.

Two LMMs were applied to each of these dependent variables. LMM1 examined the overall effects of the N-Back level and the effect of SCR on the observed t-values, whereas LMM2 estimated fixed effects for each specific combination of fNIRS channel and N-Back level. In both models, subject ID was included as a random factor “subject” to account for inter-subject variability in hemodynamic responses. Table 2 provides a summary of the fixed effects included in each LMM and their corresponding model structures.

**Table 2:** Summary of the fixed factors included in the LMMs used in the analysis. The dependent variables were the t-values from the GLM analyses with SCR (GLM-SCR) and without SCR (GLM-noSCR), calculated separately for HbO and HbR signals. LMM1 assessed the main effects of N-Back level and SSC signal amplitude. LMM2 directly modeled each unique combination of channel ID and N-Back level as a separate fixed effect, allowing estimation of condition-specific effects at each channel. In all models, the subject was included as a random effect to account for inter-subject variability.

LMM	Dependent variable	N-Back level	SSC	Channel ID * N-Back Level
1	GLM-SCR-HbO	x	x	
	GLM-noSCR-HbO	x		
	GLM-SCR-HbR	x	x	
	GLM-noSCR-HbR	x		
2	GLM-SCR-HbO			x
	GLM-noSCR-HbO			x
	GLM-SCR-HbR			x
	GLM-noSCR-HbR			x

We estimated the LMM parameters using the statsmodels.mixedlm() function with maximum likelihood (ML) estimation. Although restricted maximum likelihood (REML) is generally preferred for variance component estimation, we used ML because LMM1-SCR versus LMM1-noSCR differed in their fixed-effects structure—particularly the inclusion of SSC as a covariate. ML provides valid model comparison under such circumstances and ensures consistency across models when comparing fixed-effects estimates, even in LMM2 where the fixed-effect structure was matched across SCR conditions.^69^^,^^70^

#### 2.5.3 LMM1: load effects and effects of SSC

As mentioned above, the dependent variables were the t-values for either the HbO or HbR from GLM-SCR and GLM-noSCR. This resulted in four linear models: LMM1-SCR-HbO, LMM1-SCR-HbR, LMM1-noSCR-HbO, and LMM1-noSCR-HbR. Each model was controlled for the random factor “subject,” as mentioned above.

The fixed factor N-Back level was included in each model to investigate the impact of N-Back levels on hemoglobin concentrations, providing evidence for the hypothesis that increasing WML leads to increased HbO and decreased HbR concentrations.

In addition, by comparing the results from the models with and without SCR (LMM1-SCR-HbO versus LMM1-noSCR-HbO, LMM1-SCR-HbR versus LMM1-noSCR-HbR), we investigated the effect of SCR on measured hemodynamics to assess the hypothesis that the SCR would improve statistical results by yielding larger t-values and more significant channels.

LMM1 included SSC (ShortHbO and ShortHbR) as a fixed factor to assess how scalp-level hemodynamics influence t-values from GLM-SCR in the long channels.

To account for superficial physiological noise, the GLM-SCR included the average short-channel hemoglobin concentrations as regressors. These regressors may reflect extracerebral hemodynamics, allowing us to assess whether long channels capture both cerebral and scalp signals. An effect of the SSC factor in the LMM would thus support this hypothesis. To evaluate WML-related changes in brain activity, we performed paired t-tests comparing individual N-Back levels and quantified effect sizes using Cohen’s d. The results were then compared between LMMs with and without SCR, for both HbO and HbR, to assess the influence of SCR on detecting load-dependent hemodynamic responses.

#### 2.5.4 LMM2: load effects and effects of SSC at channel level

LMM2 included a fixed effect for each unique combination of channel ID and N-Back level to examine the joint influence of WML and channel-specific responses. This approach provided a more granular analysis of the hypotheses that higher WML would lead to increased HbO signals and decreased HbR signals and that SCR would enhance statistical outcomes at the channel level.

We conducted paired t-tests as factor level analysis to compare differences between various N-Back levels for each channel, with Cohen’s d to measure effect size. These joint effects were then compared between LMMs-SCR and LMMs-noSCR for both HbO and HbR, further evaluating the aforementioned hypotheses.

To evaluate the consistency of group-level effects at the single-subject level, we summarized the GLM results at the subject level. For each N-Back level contrast and each channel, we counted the number of subjects with a significant contrast. More specifically, at the subject level, the contrasts between N-Back levels were calculated as $t=\frac{\beta_{A}-\beta_{B}}{\sqrt{\epsilon^{2}}}$, where A and B represent different N-Back levels. Here, $\beta_{A}$ and $\beta_{B}$ are the estimated regression coefficients (activation amplitudes) for respective N-Back conditions A and B, and ϵ represents the standard error of the contrast estimate.

## 3 Results

### 3.1 Behavioral Results

**Fig. 2:**
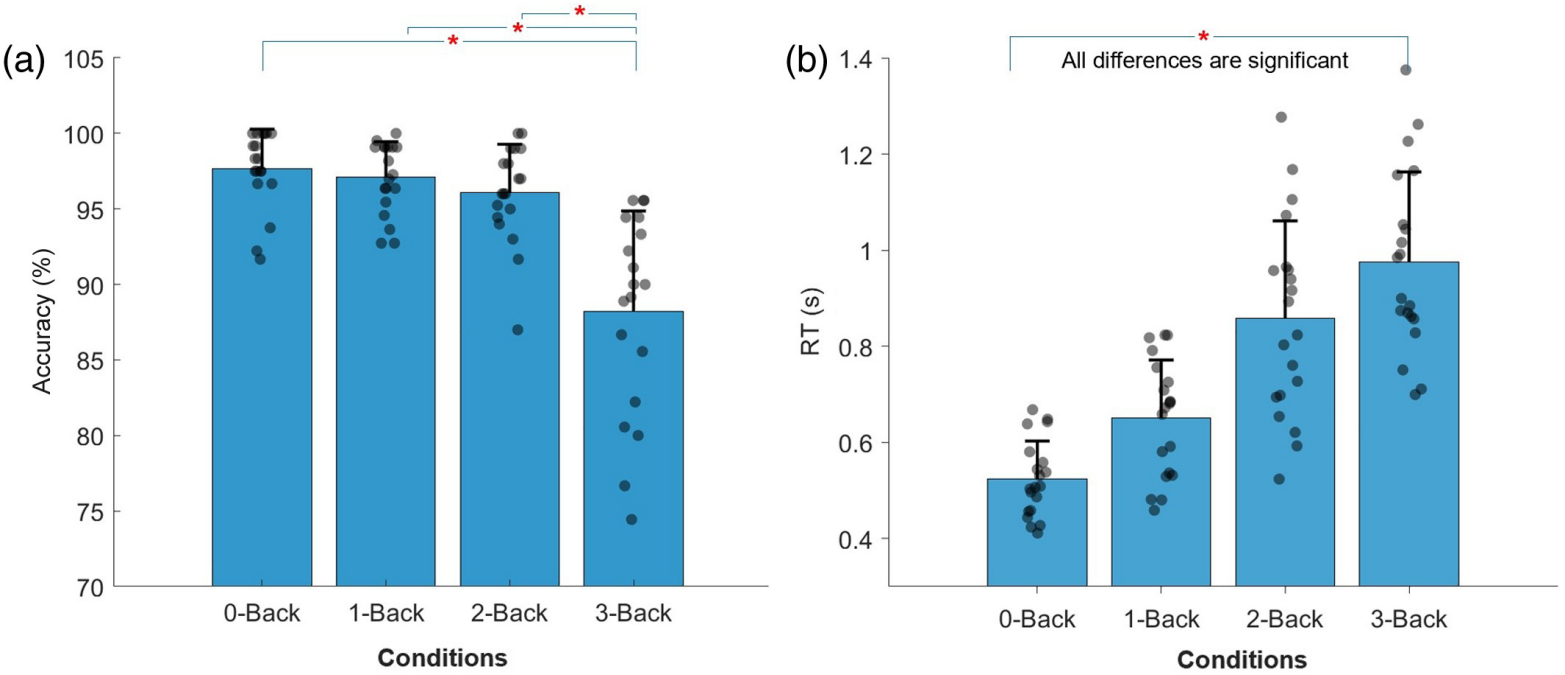
Mean response accuracy and RT for each N-Back level, n=20. Error bars represent +1 *SD*. Dots represent individual participants’ mean values per condition and are jittered horizontally for visualization. (a) The pairwise comparisons (t-tests) of accuracy in the 3-Back condition and each of the remaining conditions were significant at an α level of 0.05 after FDR correction, as indicated by “*”. Responses were less accurate with higher N-Back levels. (b) Mean RTs for each N-Back level, with error bars representing ±1 *SD*. All pairwise comparisons were significant at an α level of 0.05 after FDR correction. RTs increased with increasing N-Back level.^71^

The mean response accuracy for each N-Back level is shown in Fig. 2(a). The repeated measures ANOVA with the factor N-Back level showed a significant main effect of the N-Back level on response accuracy (F(3,76)=20.905, p<0.001, $\eta_{p}^{2}=0.913$).

As anticipated, as the N-Back level increased, indicating a higher WML, response accuracy correspondingly decreased. Post-hoc analysis of paired t-test with FDR correction showed that accuracy was significantly lower for the 3-Back level (M=0.882, SD=0.062) compared with each of the other N-Back levels (0-Back: M=0.977, SD=0.021, t(19)=5.784, p<0.001, d=1.877; 1-Back: M=0.971, SD=0.020, t(19)=5.484, d=1.779; 2-Back: M=0.961, SD=0.032, t(19)=4.637, d=1.504). No significant differences were found between the 0-Back, 1-Back, and 2-Back tasks in pairwise comparisons after adjusting for multiple comparisons.

Mean RTs for each N-Back level are displayed in Fig. 2(b). The repeated measures ANOVA of RTs showed a significant effect of the N-Back level (F(3,76)=1187.050, p<0.001, $\eta^{2}=0.278$).

As expected, the RTs varied significantly among the N-Back levels. Performance on the 0-Back (M=0.522  s, SD=0.131  s) task was significantly faster than all the other task conditions (1-Back: M=0.971  s, SD=0.020  s, t(19)=5.484, d=0.569; 2-Back: M=0.879  s, SD=0.248  s, t(19)=44.049, p<0.001, d=1.242; 3-Back: M=0.992  s, SD=0.315  s, p<0.001, t(19)=54.571, d=1.561). The 1-Back task had significantly faster RTs than the 2-Back: (p<0.001, t(19)=25.438, d=0.740) and 3-Back: (p<0.001, t(19)=35.930, d=1.066). Finally, the 2-Back task had significantly faster RTs than the 3-Back task (p<0.001, t(19)=10.108, d=0.313).

### 3.2 LMM1: Load Effects and Effects of SSC

We examined the effect of SCR on measured HbO and HbR concentration changes across different N-Back levels. The significant results for each factor in LMM1-SCR and LMM1-noSCR for both HbO and HbR are presented below, with β representing the coefficients of the fixed factors in the LMMs.

1. LMM1 fixed factors
  • LMM1-SCR: For LMM1-SCR-HbO, the 2-Back condition exhibited the largest increase in HbO channels (β=13.812, p<0.001, d=7.291), followed by 3-Back (β=10.537, p<0.001, d=5.562) and then 1-Back conditions (β=7.401, p<0.001, d=3.907), and 0-Back condition (β=6.596, p<0.001, d=3.482). The coefficients from 0-Back to 2-Back increased progressively with higher N-Back levels, suggesting that HbO increased with increased WML, which was confirmed by N-Back level contrasts as reported below. ShortHbO showed a significant effect (β=4.291, p=0.023, d=2.264) suggesting that the SSC-measured HbO signals accounted for HbO concentration changes. For LMM1-SCR-HbR, the 3-Back condition exhibited the largest decrease in HbR channels (β=−7.009, p<0.001, d=5.536), followed by the 2-Back (β=−3.296, p<0.001, d=2.603), and then, 1-Back conditions (β=1.327, p=0.295, d=1.048) without showing significance, and 0-Back condition (β=5.128, p<0.001, d=4.050). The coefficients of 0-Back to 3-Back were decreasing with increased N-Back levels, suggesting the HbR decreased with increased WML, which again was confirmed by N-Back level contrasts. ShortHbR did not show significant effects.
  • LMM1-noSCR: For LMM1-noSCR-HbO, significant changes in hemodynamic responses were obtained for the 2-Back (β=13.570, p<0.001, d=5.337) and 3-Back conditions (β=10.421, p<0.001, d=4.099), followed by 1-Back condition (β=7.118, p=.005, d=2.799) and the 0-Back condition (β=6.281, p=0.013, d=2.470). These results were similar to results with SCR yet with smaller β coefficients for each level of the fixed factor. For LMM1-noSCR-HbR, the 3-Back condition again exhibited the largest decrease in HbR channels (β=−6.798, p<0.001, d=3.949), whereas the 2-Back (β=−3.254, p=0.059, d=1.889) and 1-Back conditions (β=1.213, p=.481, d=0.704) did not reach significance, and the 0-Back condition did show a significant effect (β=5.259, p=0.002, d=3.053). Overall, the LMM1-noSCR-HbR thus showed less significant results than LMM1-SCR-HbR; and LMM1-noSCR had smaller |t-values| than LMM1-SCR for both HbO and HbR.
2. Factor level analysis

We used paired t-tests to analyze contrasts between the levels of the fixed factor N-Back level, without correcting for multiple comparisons. The results indicated that, for HbO, all contrasts except for 1-Back versus 0-Back were significant. For HbR, all contrasts were significant. Notably, the 2-Back condition showed a greater hemodynamic response than the 3-Back condition for both HbO and HbR, indicating a stronger activation under medium WML compared with high WML. Importantly, the results did not differ between LMM1 with SCR and without SCR. Details of the comparisons, including t-values, p-values, effect sizes, and significance levels, are listed in Table 3.

**Table 3:** LMM1: summary of the paired t-tests of N-Back level contrasts for measured HbO and HbR and for LMM1-SCR and LMM1-noSCR. The task-related hemodynamic changes differed significantly between the N-Back levels. In this table, a positive t-value indicates that the first N-Back level in the N-Back level contrast had a higher mean HbO or HbR than the second N-Back level (e.g., a positive t-value for 1-Back versus 2-Back means 1-Back HbO >2-Back HbO). Conversely, a negative t-value indicates that the second N-Back level had a higher mean. For HbO, all contrast between N-Back levels were significant, except for the 1-Back versus 0-Back comparison. The N-Back contrasts with significant effects were the same for LMM1-SCR and LMM1-noSCR. Similarly, for HbR, the significant effects for all N-Back levels were the same between LMM1-SCR and LMM1-noSCR.

N-Back contrast	Model	Hemoglobin	t-Value	p-Value	d	Significance
1-Back versus 0-Back	SCR	HbO	1.074	0.284	0.047	
	No-SCR	HbO	1.114	0.266	0.320	
	SCR	HbR	−4.782	<0.001	0.050	*
	No-SCR	HbR	−5.283	<0.001	0.276	*
1-Back versus 2-Back	SCR	HbO	7.986	<0.001	0.340	*
	No-SCR	HbO	8.085	<0.001	0.278	*
	SCR	HbR	−6.730	<0.001	0.353	*
	No-SCR	HbR	−6.496	<0.001	0.269	*
1-Back versus 3-Back	SCR	HbO	3.417	<0.001	0.183	*
	No-SCR	HbO	3.589	<0.001	0.529	*
	SCR	HbR	−10.554	<0.001	0.186	*
	No-SCR	HbR	−10.049	<0.001	0.507	*
2-Back versus 0-Back	SCR	HbO	8.503	<0.001	0.372	*
	No-SCR	HbO	8.557	<0.001	0.473	*
	SCR	HbR	−8.326	<0.001	0.386	*
	No-SCR	HbR	−8.497	<0.001	0.481	*
2-Back versus 3-Back	SCR	HbO	−5.579	<0.001	0.160	*
	No-SCR	HbO	−5.383	<0.001	0.199	*
	SCR	HbR	4.806	<0.001	0.158	*
	No-SCR	HbR	4.542	<0.001	0.190	*
3-Back versus 0-Back	SCR	HbO	4.504	<0.001	0.208	*
	No-SCR	HbO	4.679	<0.001	0.713	*
	SCR	HbR	−13.386	<0.001	0.224	*
	No-SCR	HbR	−13.275	<0.001	0.711	*

### 3.3 LMM2: Load Effects and Effects of SSC at the Channel Level

We investigated SCR effects on different N-Back levels at the channel level.

1. Fixed factors

For this purpose, we inspected the combination effect of Channel ID * N-Back level, separately for LLM2-SCR and LLM2-noSCR. To summarize the results, we report the number of channels with significant Channel ID * N-Back level joint effects. The significant combinations (Channel ID * N-Back level combination with a p-value<0.05) of LMM2 with SCR and without SCR were as follows:

• LMM2-SCR: For LMM2-SCR-HbO, the PFC exhibited 39 significant Channel ID * N-Back level combinations, indicating that 39 Channel ID * N-Back level combinations in the PFC showed significant changes in HbO in response to varying N-Back levels. The PPC showed 22 significant combinations for HbO. For HbR, 12 channels in the PFC and 4 channels in the PPC demonstrated significant combinations.
• LMM2-noSCR: For LMM2-noSCR-HbO, the PFC demonstrated a reduction to 36 significant Channel ID * N-Back level combinations, whereas the PPC had 20 significant combinations, showing fewer channels were affected by WML without SCR. For HbR, no changes were observed in the number of significant joint effects across brain regions compared with LMM2-SCR.

These results indicate that both the PFC and PPC were responsive to different WML levels, and that individual channels exhibited different N-Back level effects on HbO and HbR. Moreover, SCR increased the number of significant Channel ID * N-Back level combinations, suggesting enhanced sensitivity to load-dependent effects in the hemodynamic measurements, particularly for HbO.

2. Factor level analysis

We inspected contrasts between N-Back levels for each channel separately for LMM2-SCR and LMM2-noSCR, to find load-sensitive channels and SCR effects at the channel level. Paired t-tests revealed that the hemodynamic responses varied significantly among different N-Back levels. To summarize the results, we report the number of significant contrasts between each channel’s different N-Back level combinations.

• LMM2-SCR: For LMM2-SCR-HbO, we observed significant changes in HbO and HbR concentrations across 43 channel ID * N-Back level combinations. Specifically, LMM2-SCR-HbO showed 21 significant contrasts and LMM2-SCR-HbR presented 22 significant contrasts among different N-Back levels.
• LMM2-noSCR: Without SCR, the number of significant channel ID * N-Back level combinations decreased slightly to 41. Specifically, LMM2-noSCR-HbO showed 20 significant pairwise contrasts, and LMM2-noSCR-HbR exhibited 21 significant pairwise contrasts. This indicates that including SCR in the analysis enhanced the sensitivity, leading to more significant contrasts being detected between different N-Back levels.

**Fig. 3:**
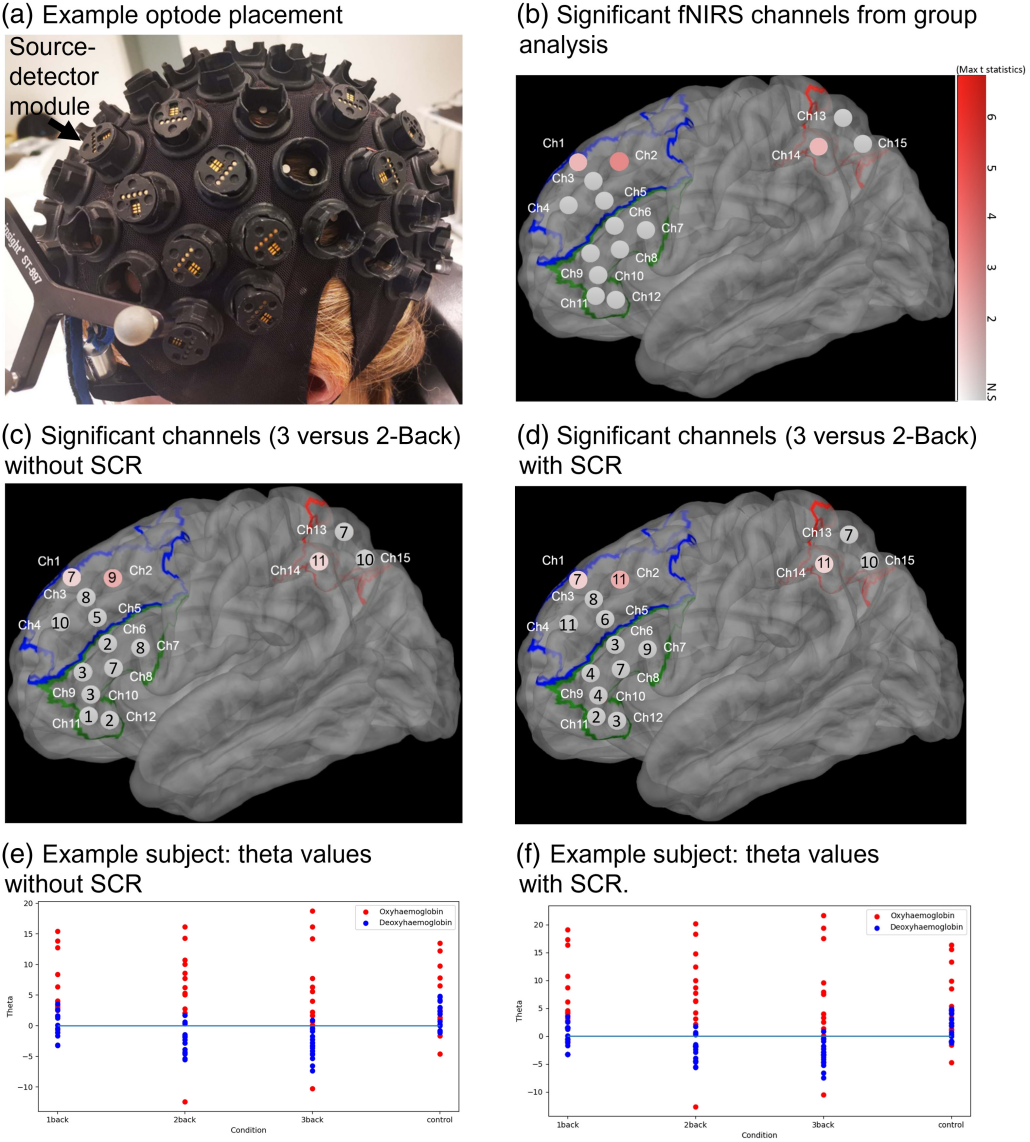
fNIRS montage and analysis visualization. Panel (a) shows a representative optode montage with embedded SSC, whereas panel (b) visualizes long-channel t-statistics, short channels are not separately shown. Panel (b) displays group-level significant channels. The dots in red shade show the positions of the significant channels that were able to discriminate between consecutive N-Back load levels. Each channel was color-coded according to the maximum t-value of consecutive load levels’ paired t-tests. Channels that did not show significant differences are represented in grey (N.S.). Panels (c) and (d) show group-level significant channels between 3 and 2-back conditions, without (c) and with (d) SCR. The same color scale is used across both maps. Panels (e) and (f) present single-subject theta (beta) maps from GLM (red for HbO and blue for HbR channels), where SCR increases contrast and signal magnitude in significant channels.

To highlight the most load-sensitive channels, we investigated channels that could distinguish between consecutive load levels, namely, 1-Back versus 2-Back, and 2-Back versus 3-Back. The comparisons between LMM2-SCR and LMM2-noSCR showed three channels that had significant N-Back contrasts among consecutive load levels, and these were all HbO channels. Specifically, Ch1 and Ch2 in the DLPFC showed a significant difference in HbO concentration between the 1-Back versus 2-Back and 2-Back versus 3-Back conditions. This pattern was also evident in Ch14 in the PPC. This ability to discriminate consecutive N-Back levels was enhanced in channels within both DLPFC and PPC with SCR. The details are shown in Table 4. Moreover, the effects for individual channels are visualized in Fig. 3(b). The figure shows all 15 long channels (channels from Table 1) on the MNI template brain. Each channel was color-coded based on its maximal t-value of its paired t-tests between consecutive load levels. Figure 3(b) shows that Ch2 had the largest t-values among all the channels that could distinguish among consecutive load levels.

**Table 4:** LMM2: Summary of paired t-test results for channels that showed significant differences in hemodynamic responses between consecutive N-Back levels (1-Back versus 2-Back and 2-Back versus 3-Back), for LMM2-SCR and LMM2-noSCR. The results were similar for LMM2-SCR and LMM2-noSCR.

N-Back contrast	Model	Hemoglobin	Channel ID	t-Value	p-Value	d
1-Back versus 2-Back	SCR	HbO	Ch1	2.371	0.033	0.710
	No-SCR	HbO	Ch1	2.441	0.030	0.729
	SCR	HbO	Ch2	3.297	0.005	0.775
	No-SCR	HbO	Ch2	3.288	0.005	0.833
	SCR	HbO	Ch14	2.476	0.029	0.526
	No-SCR	HbO	Ch14	2.474	0.029	0.516
	SCR	HbR	Ch2	−2.637	0.019	0.730
	No-SCR	HbR	Ch2	−2.636	0.019	0.729
2-Back versus 3-Back	SCR	HbO	Ch1	−2.618	0.021	0.689
	No-SCR	HbO	Ch1	−2.520	0.026	0.696
	SCR	HbO	Ch2	−2.830	0.013	0.486
	No-SCR	HbO	Ch2	−2.790	0.014	0.509
	SCR	HbO	Ch14	−2.526	0.027	0.520
	No-SCR	HbO	Ch14	−2.567	0.025	0.520

### 3.4 Subject-Level fNIRS Activation Results

We analyzed N-Back level contrast effects at the subject level across 15 channels (Ch1 to Ch15) to check the consistency of identified load-sensitive channels between group level and subject levels analysis. We analyzed subject-level GLM analysis: For each channel and each N-Back level contrast, we counted the number of subjects out of 20 for whom the respective contrast was significant. These counts are summarized in Table 5 for both GLM-SCR and GLM-noSCR. Overall, this table summarized the number of subjects with a significant effect for each combination of channel and N-Back level contrast. For each N-Back level contrast, we first identified the channels with the highest number of significant activations across subjects using GLM-SCR. We then compared these results to GLM-noSCR to evaluate the impact of SCR on detecting significant activations. The channels with the most significant activations based on the number of subjects showing significant activation across the 20 participants were as follows:

• 3-Back versus 2-Back: Ch2, Ch4, and Ch14 showed the highest activation counts (i.e., at least 11 subjects). Multiple channels (Ch2, Ch4, Ch5, Ch6, Ch7, Ch9, Ch10, Ch11, Ch12) showed reduced number of channel activation counts without SCR applied.
• 3-Back versus 1-Back: Ch1, Ch2, Ch4, and Ch14 showed the highest activation counts. Two channels (i.e., Ch4 and Ch14) showed a reduced number of channel activation counts without SCR. Three channels (i.e., Ch2, Ch3, and Ch15) showed increased activation counts without SCR.
• 3-Back versus 0-Back: Ch2, Ch4, and Ch14 showed the highest activation counts. Two channels (i.e., Ch1 and Ch9) exhibited a reduced number of activation counts and one channel (Ch15) showed increased counts without SCR.
• 2-Back versus 1-Back: Ch1, Ch2, Ch4, and Ch13 showed the highest activation counts. Four channels (i.e., Ch1, Ch2, Ch13, and Ch15) exhibited a reduced number of activation counts without SCR. A few channels (i.e., Ch3, Ch4, Ch5, Ch11, and Ch14) increased without SCR.
• 2-Back versus 0-Back: Ch1, Ch2, Ch4, Ch13, and Ch14 showed the highest activation counts. Three channels (i.e., Ch2, Ch3, and Ch15) exhibited a reduced number of activation counts without SCR. Three channels (i.e., Ch1, Ch5, and Ch14) showed increased activation counts without SCR.
• 1-Back versus 0-Back: Ch4, Ch13, and Ch14 showed the highest activation counts. Four channels (i.e., Ch8, Ch13, Ch14, and Ch15) exhibited a reduced number of activation counts without SCR. Two channels (i.e., Ch2 and Ch4) showed increased activation counts without SCR.

**Table 5:** Comparison of channel activation counts of GLM analysis across different N-Back levels with and without SCR. Counts of subjects out of 20 showing significant activation for each N-Back level in the N-Back memory task. In each cell, counts are presented as with SCR/without SCR. For each N-Back level, some channels showed different activation counts between GLM-SCR and GLM-noSCR.

Channel ID	3-Back versus 2-Back	3-Back versus 1-Back	3-Back versus 0-Back	2-Back versus 1-Back	2-Back versus 0-Back	1-Back versus 0-Back
Ch1	7/7	12/12	7/6	13/12	11/12	6/6
Ch2	11/9	11/12	11/11	14/13	12/13	9/10
Ch3	8/8	8/9	9/9	11/12	13/11	8/8
Ch4	11/10	12/11	14/14	14/15	14/14	12/13
Ch5	6/5	7/7	9/9	6/7	7/9	9/9
Ch6	3/2	1/1	2/2	1/1	2/2	2/2
Ch7	9/8	7/7	8/8	9/9	9/9	8/8
Ch8	7/7	7/7	6/6	7/7	6/6	6/5
Ch9	4/3	2/2	2/1	1/1	2/2	2/2
Ch10	4/3	3/3	3/3	4/4	4/4	3/3
Ch11	2/1	3/3	2/2	2/3	2/2	2/2
Ch12	3/2	2/2	2/2	3/3	2/2	2/2
Ch13	7/7	9/9	10/10	12/11	11/11	12/10
Ch14	11/11	13/12	13/13	10/12	12/14	11/10
Ch15	10/10	8/9	9/10	11/10	11/10	11/10

Overall, Ch2, Ch4, Ch13, and Ch14 emerged as the most responsive channels across multiple N-Back levels at the subject level, indicating their crucial role in areas of the brain engaged during the N-Back task, which is corroborated by the group-level analysis indicating these channels’ significant activations. These channels showed a consistent pattern of high activation, making it a key area for further investigation in WML studies.

## 4 Discussion

Our study sought to examine whether the effects observed in a standard cognitive task with minimal motor demands—such as the N-Back—might still be influenced by superficial physiological activity. Specifically, we investigated whether SCR can enhance the sensitivity of fNIRS measurements by mitigating scalp-related noise, thereby improving the detection of WML-dependent cortical activation. Using the N-Back task to systematically manipulate WML, we examined three hypotheses: (1) higher N-Back levels would elicit increased activation in the PFC and PPC, reflected by increased HbO and decreased HbR concentrations, (2) SCR would improve statistical sensitivity in detecting load-dependent hemodynamic changes, and (3) group-level effects would be also observed at the individual level when using SCR.

### 4.1 Hypothesis 1: Load-Dependent Activation in PFC and PPC

The first hypothesis predicted that increasing the N-Back level and, hence, the WML would result in increased PFC and PPC activity, corresponding to increased HbO and decreased HbR. Our results partially supported this hypothesis, indicating that SCR improved statistical sensitivity for detecting cerebral oxygenation changes in the N-Back task across some channels and conditions and at the individual level but not uniformly across all models. Both LMM1 and LMM2’s analyses of the N-Back factor levels showed significant activation differences among 1-Back, 2-Back, and 3-Back levels. For example, in the 2-Back condition, both LMM1-SCR-HbO and LMM1-noSCR-HbO showed greater task-evoked frontoparietal activity compared with the 1-Back and 0-Back conditions, whereas the 3-Back task, in comparison to the 2-Back task, exhibited nonmonotonic activation patterns. These findings align with earlier fNIRS research,^29^^,^^72^^–^^74^ which also observed increasing HbO concentration levels with N-Back up to 2-Back, followed by a nonmonotonic decrease from 3-Back compared with 2-Back. Similarly, for LMM1-SCR-HbR and LMM1-noSCR-HbR, HbR signals showed a monotonic decrease with a higher N-Back level. In LMM2, the channel-wise analysis showed a similar activation pattern, which also suggested WML-dependent hemodynamic response changes.

Furthermore, subject-level patterns mirrored the group-level activation shown by LMM1 and LMM2 as well. Specifically, Ch2 (middle frontal gyrus) and Ch14 (inferior parietal cortex) emerged as the most responsive channels across several contrasts, with higher brain activation when the WML was increased up to 2-Back. Previous fNIRS studies have also found both linear and nonlinear increases in frontal activity with greater N-Back levels.^36^^,^^75^^,^^76^ Nonmonotonic effects, in which increased WML does not always result in proportionately higher brain activity, are hypothesized to occur when task difficulty causes disengagement or when peak cortical activation is reached.^51^^,^^72^ The results of our research partially replicate fMRI studies^47^^,^^48^ showing that the measured hemodynamics regarding HbO signals increased monotonically up to the 2-Back condition, but for the 3-Back condition, the changes in HbO concentration decreased.

### 4.2 Hypothesis 2: Effects of SCR on Statistical Sensitivity

Our second hypothesis proposed that using SCR would improve the sensitivity of cortical activation measurements by reducing scalp hemodynamic interference. This hypothesis was confirmed. At the group level, both LMM1 and LMM2 demonstrated that the use of SCR led to an increase in t-values for the coefficients of the fixed factors, indicating improved sensitivity in detecting hemodynamic responses. However, the overall changes in the coefficients between models with and without SCR were modest, suggesting that the underlying hemodynamic responses in the brain remained consistent. The inclusion of SCR primarily enhanced the measurement of these effects. Specifically, LMM1 showed that models using SCR resulted in stronger effects, as evidenced by higher coefficients and increased t-values in the factor level paired t-tests of N-Back levels. In addition, LMM1-SCR-HbR had more significant fixed factor levels than LMM1-noSCR-HbR.

Moreover, in LMM1-SCR-HbR and LMM1-noSCR-HbR, the factor-level analyses revealed significant differences between the 1-Back and 0-Back levels. By contrast, no such effect was observed for HbO in either LMM1-SCR-HbO or LMM1-noSCR-HbO. Interestingly, when examining the fixed effects of the N-Back factor, the pattern reversed: significant effects were detected in the HbO models but not in the HbR models. This discrepancy between HbO and HbR may reflect their differential susceptibility to extracerebral confounds. Prior studies have shown that HbR is generally less affected by superficial physiological noise,^53^^,^^55^ making its signal more robust across preprocessing conditions. Conversely, HbO is more vulnerable to systemic physiological fluctuations, such as those induced by WML (e.g., heart rate or blood pressure changes), which may vary across N-Back levels. SCR appears to effectively mitigate these confounding effects in HbO signals, enhancing sensitivity to true cortical activation. Taken together, these results suggest that the added value of SCR is particularly pronounced for HbO-based analyses in cognitive paradigms, especially those involving WML.

### 4.3 Hypothesis 3: Individual-Level Consistency

Although group-level improvements with SCR were moderate, individual-level analyses revealed some consistent patterns. Moreover, in LMM1-SCR, the SSC effect was significant, which indicated that measured hemodynamics contained scalp hemodynamic responses. In LMM2, incorporating SCR improved the sensitivity of recognizing task-related activations, resulting in more significant Channel ID * N-Back level pairs. At the subject level, comparing data with and without SCR for each channel also indicated differences in channels’ activation detection. As visible in Table 5, SCR altered the number of participants showing significant activation in several channels, particularly under higher WML conditions such as the 3-Back versus 2-Back contrast, which represents the most cognitively demanding transition in our task. These results suggest that SCR enhanced the detection of cognitive effects in some individuals by reducing scalp-related noise, thereby improving the robustness and sensitivity of subject-specific hemodynamic responses. Overall, our channel-wise results at the subject level showed consistency with group-level results, and differences between load levels were able to be detected even at the single subject level, pointing to the potential for developing subject-level decoding models. Further research could aim to train machine learning-based decoding models to decode WML at the subject level.

Our findings were consistent with and expanded prior research that emphasized the relevance of addressing extracerebral hemodynamics in fNIRS data for motor tasks such as finger tapping^25^^,^^26^^,^^51^ as well as auditory tasks.^27^^,^^28^ Our study also expanded on previous research by demonstrating the usefulness of SCR in a basic WM experiment for assessing WML. Zhou et al.^27^ and Wyser et al.^25^ observed that SCR did not significantly improve results compared with nonSCR approaches. Nevertheless, their findings implied that although SCR could boost the t-values and potentially improve the statistical significance of particular measures, the overall influence on the results might be moderate. This conclusion is consistent with our findings as LMM2 channel-level analysis revealed more significant pairs and load contrasts with SCR, whereas the differences were modest. In our findings, SCR application led to greater coefficients in the fixed factors of LMMs and t-values from factor-level paired t-test, in agreement with Wyser et al.’s findings.^25^ They also emphasized that, although SCR might not have a substantial impact on categorization accuracy in brain–computer interface settings, it could be still beneficial for applications examining the origin, patterns, or magnitudes of brain activity. Our findings demonstrate that SCR enhances results even in cognitive tasks with minimal motor requirements. This suggests that SCR can reveal effects with a given effect size in shorter experiment times or with fewer participants, even in the absence of significant motor confounds as it is usually the case in cognitive experiments. Our findings have important implications for both research and applied applications of fNIRS. We demonstrated that SCR improved fNIRS results, allowing researchers to better analyze basic cognitive task-related cortical activity patterns.

Despite promising outcomes, significant questions and limitations still remain. First, during piloting, we explored discarding the initial 2 s of each task block to reduce potential hemodynamic transition effects. However, this approach resulted in lower statistical sensitivity across several participants. As a result, the full block duration was retained for analysis. Notably, Sunwoo et al. employed a more extended 10-s discard period to account for onset-related noise, which may be beneficial in longer or more variable paradigms. Future work should carefully balance signal quality with effect detectability when handling block onsets. Second, although SCR generally improved the interpretability of hemodynamic responses, its added value was modest in our study. Nevertheless, our individual-level analysis revealed subtle but consistent differences in activation patterns, especially in more cognitively demanding contrasts such as 3-Back versus 2-Back. These results suggest that SCR may enhance subject-level sensitivity, even when group-level improvements are less pronounced—an insight particularly relevant for personalized or clinical applications of fNIRS. Last, several optode batteries failed over the course of data collection. As a result, the number and placement of functional channels varied across participants, potentially limiting the uniformity of ROI coverage and reducing the power to detect consistent activation patterns across the sample.

Moreover, the development of more advanced signal processing techniques to optimize the separation of cerebral signals from extracerebral noise may further enhance the utility of fNIRS. Combining SCR with other technologies, such as machine learning algorithms, may result in even more effective noise reduction and improved cortical activation estimations.

## 5 Conclusion

Our findings demonstrate that SCR can modestly improve the sensitivity and validity of fNIRS in working memory tasks, particularly for HbO signals and at the individual level. We observed robust load-dependent activation up to the 2-Back level in the DLPFC and PPC, with the middle frontal gyrus showing the strongest response. These results suggest that even in paradigms with minimal motor requirements, SCR can still enhance the sensitivity and validity of fNIRS measurements. Future research should refine SCR approaches, test their utility across cognitive domains, and explore their potential in clinical and real-world applications of fNIRS.

## Data Availability

The data and code supporting the study’s conclusions are accessible from the corresponding author upon reasonable request.
